# 3,5-Bis(benz­yloxy)benzoic acid

**DOI:** 10.1107/S1600536812043796

**Published:** 2012-10-31

**Authors:** Rodolfo Moreno-Fuquen, Carlos Grande, Rigoberto C. Advincula, Juan C. Tenorio, Javier Ellena

**Affiliations:** a Departamento de Química, Facultad de Ciencias, Universidad del Valle, Apartado 25360, Santiago de Cali, Colombia; b Programa de Ingenieria Agroindustrial, Universidad San Buenaventura, AA 7154, Santiago de Cali, Colombia; c Case Western Reserve University, Department of Macromolecular Science and Engineering, 2100 Adelbert Road, Kent Hale Smith Bldg, Cleveland, Ohio 44106, USA; d Instituto de Física de São Carlos, IFSC, Universidade de São Paulo, USP, São Carlos, SP, Brazil

## Abstract

In the title compound, C_21_H_18_O_4_, the outer benzyl rings are disordered over two resolved positions in a 0.50 ratio. The O—CH_2_ groups form dihedral angles of 4.1 (2) and 10.9 (4)° with the central benzene ring, adopting a *syn–anti* conformation with respect to this ring. In the crystal, the mol­ecules are linked by O—H⋯O hydrogen bonds and weak C—H⋯O inter­actions, forming chains along [010].

## Related literature

For properties of dendrimer chemistry, see: Fréchet (2002 ▶). For the diverse applications of 3,5-bis­(benz­yloxy)benzoic acid and its benzoate derivatives, see: Sivakumar *et al.* (2010 ▶); Remya *et al.* (2008 ▶); Hawker & Fréchet (1992 ▶). For magnetic and luminiscent properties of lanthanide benzoates, see: Busskamp *et al.* (2007 ▶). For the conformation of O—CH_2_ groups, see: Xiao *et al.* (2007 ▶). For related structures, see: Gainsford *et al.* (2009 ▶); Zhu *et al.* (2009 ▶). For graph-set motifs, see: Etter (1990 ▶). For hydrogen bonding, see: Nardelli (1995 ▶); Desiraju & Steiner (1999 ▶).
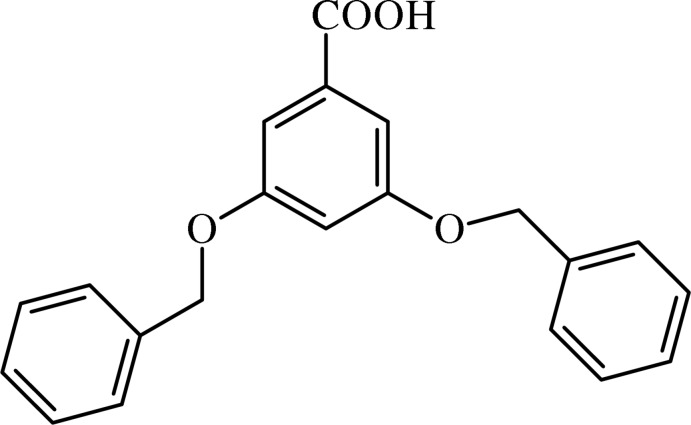


## Experimental

### 

#### Crystal data

C_21_H_18_O_4_
*M* *_r_* = 334.37
Triclinic, 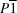

*a* = 5.2801 (2) Å
*b* = 11.6830 (5) Å
*c* = 14.4803 (7) Å
α = 83.303 (2)°
β = 80.775 (2)°
γ = 79.031 (1)°
*V* = 862.17 (6) Å^3^
*Z* = 2
Mo *K*α radiation
μ = 0.09 mm^−1^
*T* = 295 K
0.43 × 0.11 × 0.10 mm

#### Data collection

Nonius KappaCCD diffractometer
5626 measured reflections
3084 independent reflections
1801 reflections with *I* > 2σ(*I*)
*R*_int_ = 0.036

#### Refinement

*R*[*F* ^2^ > 2σ(*F* ^2^)] = 0.053
*wR*(*F* ^2^) = 0.163
*S* = 1.03
3084 reflections
316 parameters
H-atom parameters constrained
Δρ_max_ = 0.15 e Å^−3^
Δρ_min_ = −0.15 e Å^−3^

### 

Data collection: *COLLECT* (Nonius, 2000 ▶); cell refinement: *SCALEPACK* (Otwinowski & Minor, 1997 ▶); data reduction: *DENZO* (Otwinowski & Minor, 1997 ▶) and *SCALEPACK*; program(s) used to solve structure: *SHELXS97* (Sheldrick, 2008 ▶); program(s) used to refine structure: *SHELXL97* (Sheldrick, 2008 ▶); molecular graphics: *ORTEP-3 for Windows* (Farrugia, 1997 ▶) and *Mercury* (Macrae *et al.*, 2006 ▶); software used to prepare material for publication: *WinGX* (Farrugia, 1999 ▶).

## Supplementary Material

Crystal structure: contains datablock(s) I, global. DOI: 10.1107/S1600536812043796/hg5257sup1.cif

Structure factors: contains datablock(s) I. DOI: 10.1107/S1600536812043796/hg5257Isup2.hkl

Supplementary material file. DOI: 10.1107/S1600536812043796/hg5257Isup3.cml

Additional supplementary materials:  crystallographic information; 3D view; checkCIF report

## Figures and Tables

**Table 1:** Hydrogen-bond geometry (Å, °)

*D*—H⋯*A*	*D*—H	H⋯*A*	*D*⋯*A*	*D*—H⋯*A*
O2—H2⋯O1^i^	0.82	1.82	2.6333 (18)	175
C20—H20⋯O1^ii^	0.93	2.66	3.507 (13)	153

Symmetry codes: (i) 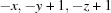; (ii) 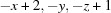.

**Fig. 1.**
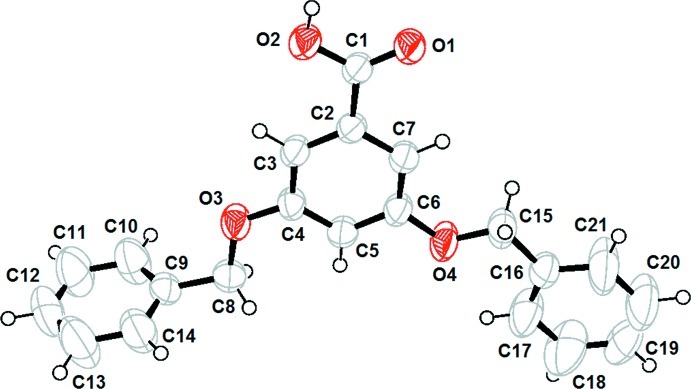
An ORTEP-3 (Farrugia, 1997) plot of (I) with displacement ellipsoids drawn at the 50% probability level. H atoms are shown as spheres of arbitrary radius.

**Fig. 2.**
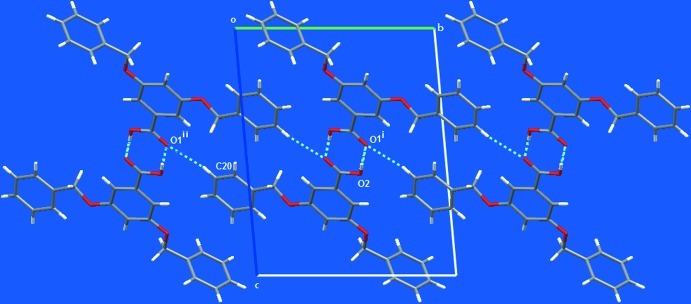
Part of the crystal structure of (I), showing the formation of chains running along [010]. Symmetry code: (i) -x,-y + 1,-z + 1; (ii) -x + 2,-y,-z + 1

## supplementary crystallographic information

### Comment

Dendrimer chemistry provides new opportunities of research in design of supramolecular architectures (Fréchet, 2002). 3,5-Bis-benzyloxy-benzoic acid (I) was used for the synthesis of luminescent lanthanide coordination complexes that display unique line-like emission bands (Sivakumar *et al.*, 2010; Remya *et al.*, 2008). Lanthanide benzoates and their derivatives have potential applications in a wide variety of fields because their novel luminescent and magnetic properties (Busskamp *et al.*, 2007). The title compound was also used in the synthesis of monodispersed dendritic polyesters with removable chain ends using a convergent growth process (Hawker & Fréchet, 1992). Other related compounds were crystallized and studied by X-ray diffraction (Gainsford *et al.*, 2009; Zhu *et al.*, 2009) and their parameters can be used to compare with the parameters of the title system. A perspective view of the molecule of (I), showing the atomic numbering scheme, is given in Fig. 1. The title compound crystallizes in the triclinic system with a P-1 space group. The outer benzyl rings are disordered over two resolved positions in a 0.50 ratio. The molecules are bonded by intermolecular O—H···O hydrogen bonds of moderate character (Desiraju & Steiner, 1999). Indeed, carbonylic O2 and O1 are linked with an O···O distance of 2.633 (2) Å. The propagation of these interactions generate centrosymmetric rings with graphs-set notation *R*_2_^2^(8) (Etter, 1990). Other weak C—H···O intermolecular interactions (Nardelli, 1995) contribute to stabilization of the molecules along b (Fig. 2). Other classical hydrogen bond interactions are not exhibited in the crystal packing. In the title structure, the O—CH_2_ groups adopt a *syn-anti* conformation with respect to the central phenyl ring, similar to the behavior presented in the 1,3-Dibenzyloxy-5-(bromomethyl)-benzene system (Zhu *et al.*, 2009), while in other similar structures, the O—CH_2_ groups adopt a *syn-syn* conformation (Xiao *et al.*, 2007). The O—CH_2_ groups, C4—O3—C8—C9 and C6—O4—C15—C16 are essentially planar (r.m.s. deviation of non-hydrogen atoms= 0.0355 Å and 0.0217 Å respectivelly) and form dihedral angles of 4.1 (2)° and 10.9 (4)° with the central phenyl ring.

### Experimental

Methyl 3,5-dihydroxybenzoate (2.0g, 12 mmol) was dissolved in 50 ml of acetonitrile and refluxed with potassium carbonate (8.0 g, 58 mmol) for 30 min. The resulting reaction mixture was refluxed at 68° C for 48 h following the addition of benzyl bromide (4.0 g, 24 mmol). The acetonitrile was evaporated off, and the residual mixture was poured into ice cold water. Methyl 3,5-bis-(benzyloxy)benzoate was obtained as a white precipitate. (2.0 g, 5.74 mmol), were taken from the precipitate, which was dissolved in 50 ml of ethanol. To this solution was added (1 g, 17.77 mmol) of KOH and it was placed under reflux. The reaction was followed by TLC until the presence of KOH was not longer observed. The reaction mixture was poured into ice cold water, acidified with dilute HCl, and the resulting precipitate was filtered, washed, dried, and recrystallized from ethanol. Yield, 1.67 g (89%). 3,5-Bis(benzyloxy)benzoic Acid, ^1^H-NMR (500 MHz) δ(p.p.m.), 7.45–7.46(d, 4H, J= 7 Hz), 7.39–7.41 (t, 4H, J=7 Hz), 7.32–7.35 (t, 2H, J= 7 Hz), 7.16(d, 2H, J= 2.5 Hz), 6.92–6.93 (t, 1H, J= 2.5 Hz), 5.15 (s, 4H). ^13^C-NMR: 166.88, 159.35, 136.64, 132.79, 138.45, 127.90, 127.63, 107.96, 106.50, 69.45. F T—IR (KBr): 3033 (Ar—H); 1690, 1159, 733, 698 cm^-1^.

### Refinement

All H-atoms were positioned geometrically using riding model with [C—H= 0.93 Å for aromatic, C—H= 0.82 Å for hydroxyl and C—H= 0.97 Å for methylene H atoms. *U*_iso_(H)= 1.2*U*_eq_(C) for aryl and methylene H atoms and 1.5*U*_eq_(O) for hydroxyl H-atom]. During the structure determination disordered sites around the two benzyl groups were found. Trial refinements were used with the split-atom approach for these extra sites with a constrained 50% occupancy each.

### Crystal data

**Table:**

C_21_H_18_O_4_	*Z* = 2
*M**_r_* = 334.37	*F*(000) = 352
Triclinic, *P*1	*D*_x_ = 1.288 Mg m^−^^3^
Hall symbol: -P 1	Mo *K*α radiation, λ = 0.71073 Å
*a* = 5.2801 (2) Å	Cell parameters from 3147 reflections
*b* = 11.6830 (5) Å	θ = 2.9–26.4°
*c* = 14.4803 (7) Å	µ = 0.09 mm^−^^1^
α = 83.303 (2)°	*T* = 295 K
β = 80.775 (2)°	Block, colourless
γ = 79.031 (1)°	0.43 × 0.11 × 0.10 mm
*V* = 862.17 (6) Å^3^	

### Data collection

**Table:**

Nonius KappaCCD diffractometer	1801 reflections with *I* > 2σ(*I*)
Radiation source: fine-focus sealed tube	*R*_int_ = 0.036
Graphite monochromator	θ_max_ = 25.2°, θ_min_ = 3.5°
CCD rotation images, thick slices scans	*h* = −6→6
5626 measured reflections	*k* = −14→14
3084 independent reflections	*l* = −17→17

### Refinement

**Table:**

Refinement on *F*^2^	Primary atom site location: structure-invariant direct methods
Least-squares matrix: full	Secondary atom site location: difference Fourier map
*R*[*F*^2^ > 2σ(*F*^2^)] = 0.053	Hydrogen site location: inferred from neighbouring sites
*wR*(*F*^2^) = 0.163	H-atom parameters constrained
*S* = 1.03	*w* = 1/[σ^2^(*F*_o_^2^) + (0.094*P*)^2^ + 0.0032*P*] where *P* = (*F*_o_^2^ + 2*F*_c_^2^)/3
3084 reflections	(Δ/σ)_max_ < 0.001
316 parameters	Δρ_max_ = 0.15 e Å^−^^3^
0 restraints	Δρ_min_ = −0.15 e Å^−^^3^

### Special details

**Table:**

Geometry. All e.s.d.'s (except the e.s.d. in the dihedral angle between two l.s. planes) are estimated using the full covariance matrix. The cell e.s.d.'s are taken into account individually in the estimation of e.s.d.'s in distances, angles and torsion angles; correlations between e.s.d.'s in cell parameters are only used when they are defined by crystal symmetry. An approximate (isotropic) treatment of cell e.s.d.'s is used for estimating e.s.d.'s involving l.s. planes.
Refinement. Refinement of *F*^2^ against ALL reflections. The weighted *R*-factor *wR* and goodness of fit *S* are based on *F*^2^, conventional *R*-factors *R* are based on *F*, with *F* set to zero for negative *F*^2^. The threshold expression of *F*^2^ > σ(*F*^2^) is used only for calculating *R*-factors(gt) *etc*. and is not relevant to the choice of reflections for refinement. *R*-factors based on *F*^2^ are statistically about twice as large as those based on *F*, and *R*- factors based on ALL data will be even larger.

### Fractional atomic coordinates and isotropic or equivalent isotropic displacement parameters (Å^2^)

**Table:**

	*x*	*y*	*z*	*U*_iso_*/*U*_eq_	Occ. (<1)
C1	0.2556 (3)	0.46372 (17)	0.58387 (13)	0.0623 (5)	
C2	0.4505 (3)	0.43437 (15)	0.65014 (12)	0.0597 (5)	
C3	0.4690 (3)	0.51344 (17)	0.71085 (12)	0.0644 (5)	
H3	0.3589	0.5858	0.7109	0.077*	
C4	0.6534 (3)	0.48473 (16)	0.77226 (12)	0.0639 (5)	
C5	0.8181 (4)	0.37791 (17)	0.77144 (13)	0.0673 (5)	
H5	0.9413	0.3585	0.8125	0.081*	
C6	0.7989 (4)	0.29977 (17)	0.70920 (14)	0.0690 (5)	
C7	0.6163 (3)	0.32636 (17)	0.64802 (13)	0.0690 (5)	
H7	0.6042	0.2734	0.6064	0.083*	
C8	0.8524 (4)	0.55033 (19)	0.88979 (15)	0.0773 (6)	
H8A	1.0236	0.5321	0.8534	0.093*	
H8B	0.8282	0.4854	0.9366	0.093*	
C9	0.8300 (5)	0.65976 (19)	0.93627 (17)	0.0705 (6)	0.50
C11	0.592 (6)	0.802 (3)	1.0445 (16)	0.132 (8)	0.50
H11	0.4429	0.8290	1.0849	0.159*	0.50
C10	0.626 (5)	0.699 (2)	0.9975 (15)	0.112 (6)	0.50
H10	0.4933	0.6553	1.0109	0.134*	0.50
C12	0.798 (6)	0.854 (3)	1.0238 (16)	0.128 (9)	0.50
H12	0.7988	0.9192	1.0546	0.153*	0.50
C13	1.003 (4)	0.8186 (14)	0.9621 (15)	0.109 (4)	0.50
H13	1.1409	0.8600	0.9478	0.131*	0.50
C14	1.007 (3)	0.7216 (16)	0.9208 (10)	0.089 (4)	0.50
H14	1.1510	0.6978	0.8771	0.107*	0.50
C15	1.0002 (5)	0.1234 (2)	0.64080 (18)	0.0965 (8)	
H15A	1.0431	0.1663	0.5806	0.116*	
H15B	0.8372	0.0966	0.6407	0.116*	
O1	0.2389 (2)	0.39389 (12)	0.52819 (9)	0.0771 (4)	
O2	0.1076 (2)	0.56551 (11)	0.58910 (9)	0.0786 (5)	
H2	0.0056	0.5746	0.5506	0.118*	
O3	0.6562 (3)	0.56890 (12)	0.82994 (9)	0.0821 (5)	
O4	0.9731 (3)	0.19714 (13)	0.71375 (11)	0.0918 (5)	
C16	1.2137 (4)	0.01969 (19)	0.65555 (17)	0.0819 (6)	0.50
C17	1.311 (3)	−0.0007 (14)	0.7341 (13)	0.123 (6)	0.50
H17	1.2647	0.0503	0.7812	0.148*	0.50
C18	1.498 (2)	−0.1088 (13)	0.7431 (10)	0.118 (4)	0.50
H18	1.5568	−0.1319	0.8008	0.141*	0.50
C19	1.592 (4)	−0.1779 (19)	0.672 (2)	0.107 (7)	0.50
H19	1.7133	−0.2461	0.6796	0.128*	0.50
C20	1.506 (2)	−0.1445 (10)	0.5925 (11)	0.119 (4)	0.50
H20	1.5708	−0.1903	0.5427	0.143*	0.50
C21	1.3231 (14)	−0.0455 (7)	0.5771 (6)	0.102 (2)	0.50
H21	1.2735	−0.0222	0.5180	0.123*	0.50
C21A	1.388 (3)	0.0108 (12)	0.7188 (12)	0.087 (4)	0.50
H21A	1.3649	0.0714	0.7574	0.105*	0.50
C17A	1.2212 (16)	−0.0818 (6)	0.6129 (5)	0.091 (2)	0.50
H17A	1.0879	−0.0863	0.5792	0.110*	0.50
C18A	1.420 (2)	−0.1752 (10)	0.6191 (9)	0.104 (3)	0.50
H18A	1.4344	−0.2395	0.5851	0.124*	0.50
C19A	1.604 (4)	−0.170 (2)	0.680 (2)	0.116 (9)	0.50
H19A	1.7397	−0.2329	0.6857	0.139*	0.50
C20A	1.588 (3)	−0.0765 (13)	0.7298 (13)	0.109 (4)	0.50
H20A	1.7085	−0.0737	0.7693	0.131*	0.50
C11A	0.945 (5)	0.8536 (15)	0.9366 (15)	0.122 (5)	0.50
H11A	1.0322	0.9136	0.9086	0.146*	0.50
C9A	0.8300 (5)	0.65976 (19)	0.93627 (17)	0.0705 (6)	0.50
C10A	0.986 (4)	0.7480 (16)	0.8932 (12)	0.101 (4)	0.50
H10A	1.1073	0.7352	0.8397	0.121*	0.50
C12A	0.777 (5)	0.870 (2)	1.021 (2)	0.118 (8)	0.50
H12A	0.7512	0.9388	1.0496	0.141*	0.50
C14A	0.663 (4)	0.678 (2)	1.0200 (13)	0.081 (3)	0.50
H14A	0.5643	0.6216	1.0477	0.097*	0.50
C13A	0.645 (4)	0.776 (2)	1.0596 (13)	0.096 (5)	0.50
H13A	0.5398	0.7840	1.1172	0.115*	0.50
C16A	1.2137 (4)	0.01969 (19)	0.65555 (17)	0.0819 (6)	0.50

### Atomic displacement parameters (Å^2^)

**Table:**

	*U*^11^	*U*^22^	*U*^33^	*U*^12^	*U*^13^	*U*^23^
C1	0.0606 (10)	0.0610 (12)	0.0628 (11)	−0.0002 (9)	−0.0110 (9)	−0.0101 (9)
C2	0.0577 (10)	0.0599 (12)	0.0602 (11)	−0.0032 (8)	−0.0116 (8)	−0.0069 (9)
C3	0.0648 (11)	0.0612 (12)	0.0641 (11)	0.0030 (8)	−0.0142 (9)	−0.0097 (9)
C4	0.0703 (11)	0.0595 (12)	0.0620 (11)	−0.0017 (9)	−0.0167 (9)	−0.0125 (9)
C5	0.0692 (11)	0.0666 (13)	0.0666 (12)	0.0001 (9)	−0.0224 (9)	−0.0100 (10)
C6	0.0713 (11)	0.0596 (12)	0.0744 (12)	0.0063 (9)	−0.0215 (10)	−0.0127 (10)
C7	0.0713 (11)	0.0648 (13)	0.0712 (12)	0.0012 (10)	−0.0203 (9)	−0.0145 (9)
C8	0.0778 (12)	0.0754 (14)	0.0830 (14)	−0.0039 (10)	−0.0320 (11)	−0.0114 (11)
C9	0.0755 (13)	0.0689 (13)	0.0719 (14)	−0.0086 (12)	−0.0259 (12)	−0.0111 (11)
C11	0.158 (16)	0.098 (11)	0.134 (13)	−0.007 (10)	0.007 (10)	−0.043 (10)
C10	0.144 (13)	0.095 (10)	0.103 (12)	−0.048 (9)	−0.001 (8)	−0.017 (8)
C12	0.20 (2)	0.100 (12)	0.089 (10)	−0.025 (10)	−0.011 (10)	−0.054 (8)
C13	0.111 (6)	0.079 (8)	0.146 (14)	−0.022 (6)	−0.025 (7)	−0.022 (7)
C14	0.090 (4)	0.078 (7)	0.096 (10)	−0.009 (4)	−0.006 (6)	−0.018 (6)
C15	0.1051 (16)	0.0757 (16)	0.1102 (18)	0.0201 (12)	−0.0423 (14)	−0.0365 (13)
O1	0.0803 (9)	0.0747 (9)	0.0783 (9)	0.0080 (7)	−0.0301 (7)	−0.0240 (7)
O2	0.0789 (8)	0.0692 (9)	0.0882 (10)	0.0108 (7)	−0.0338 (7)	−0.0184 (7)
O3	0.0962 (10)	0.0701 (9)	0.0839 (9)	0.0106 (7)	−0.0419 (8)	−0.0244 (7)
O4	0.1028 (10)	0.0712 (10)	0.1016 (11)	0.0268 (8)	−0.0480 (8)	−0.0317 (8)
C16	0.0851 (14)	0.0636 (14)	0.0955 (17)	0.0050 (11)	−0.0223 (13)	−0.0177 (12)
C17	0.118 (12)	0.134 (9)	0.086 (5)	0.055 (7)	−0.017 (7)	0.000 (5)
C18	0.134 (10)	0.096 (9)	0.104 (5)	0.034 (7)	−0.035 (7)	0.007 (6)
C19	0.131 (12)	0.051 (9)	0.136 (13)	0.002 (7)	−0.035 (11)	−0.007 (8)
C20	0.116 (7)	0.079 (7)	0.158 (11)	0.010 (5)	−0.011 (6)	−0.051 (6)
C21	0.107 (5)	0.080 (5)	0.120 (6)	0.019 (4)	−0.032 (4)	−0.041 (4)
C21A	0.082 (6)	0.072 (4)	0.107 (9)	0.013 (4)	−0.023 (5)	−0.037 (5)
C17A	0.112 (5)	0.066 (4)	0.096 (5)	−0.002 (3)	−0.026 (4)	−0.011 (3)
C18A	0.137 (9)	0.054 (5)	0.115 (6)	−0.002 (5)	−0.022 (6)	−0.007 (4)
C19A	0.090 (9)	0.087 (15)	0.138 (14)	0.036 (8)	0.012 (11)	0.002 (9)
C20A	0.087 (6)	0.082 (7)	0.158 (9)	−0.002 (5)	−0.038 (6)	−0.007 (6)
C11A	0.173 (15)	0.083 (10)	0.123 (10)	−0.058 (9)	−0.027 (9)	−0.006 (7)
C9A	0.0755 (13)	0.0689 (13)	0.0719 (14)	−0.0086 (12)	−0.0259 (12)	−0.0111 (11)
C10A	0.143 (9)	0.094 (9)	0.076 (7)	−0.058 (6)	−0.004 (5)	−0.011 (6)
C12A	0.127 (10)	0.082 (8)	0.162 (19)	−0.026 (7)	−0.043 (11)	−0.042 (8)
C14A	0.098 (5)	0.088 (9)	0.059 (6)	−0.027 (5)	−0.002 (4)	−0.017 (5)
C13A	0.109 (7)	0.112 (15)	0.069 (4)	−0.025 (8)	0.001 (5)	−0.027 (6)
C16A	0.0851 (14)	0.0636 (14)	0.0955 (17)	0.0050 (11)	−0.0223 (13)	−0.0177 (12)

### Geometric parameters (Å, º)

**Table:**

C1—O1	1.236 (2)	C15—H15B	0.9700
C1—O2	1.296 (2)	O2—H2	0.8200
C1—C2	1.484 (2)	C16—C17	1.302 (18)
C2—C3	1.374 (2)	C16—C21	1.424 (8)
C2—C7	1.393 (2)	C17—C18	1.45 (2)
C3—C4	1.391 (2)	C17—H17	0.9300
C3—H3	0.9300	C18—C19	1.36 (3)
C4—O3	1.366 (2)	C18—H18	0.9300
C4—C5	1.379 (3)	C19—C20	1.30 (4)
C5—C6	1.383 (3)	C19—H19	0.9300
C5—H5	0.9300	C20—C21	1.379 (14)
C6—O4	1.367 (2)	C20—H20	0.9300
C6—C7	1.381 (3)	C21—H21	0.9300
C7—H7	0.9300	C21A—C20A	1.33 (2)
C8—O3	1.425 (2)	C21A—H21A	0.9300
C8—C9	1.490 (3)	C17A—C18A	1.369 (14)
C8—H8A	0.9700	C17A—H17A	0.9300
C8—H8B	0.9700	C18A—C19A	1.42 (4)
C9—C14	1.26 (2)	C18A—H18A	0.9300
C9—C10	1.33 (3)	C19A—C20A	1.36 (3)
C11—C12	1.32 (5)	C19A—H19A	0.9300
C11—C10	1.41 (4)	C20A—H20A	0.9300
C11—H11	0.9300	C11A—C12A	1.40 (3)
C10—H10	0.9300	C11A—C10A	1.41 (3)
C12—C13	1.32 (3)	C11A—H11A	0.9300
C12—H12	0.9300	C10A—H10A	0.9300
C13—C14	1.34 (3)	C12A—C13A	1.42 (4)
C13—H13	0.9300	C12A—H12A	0.9300
C14—H14	0.9300	C14A—C13A	1.32 (3)
C15—O4	1.413 (3)	C14A—H14A	0.9300
C15—C16	1.510 (3)	C13A—H13A	0.9300
C15—H15A	0.9700		
			
O1—C1—O2	123.33 (16)	C16—C15—H15A	109.8
O1—C1—C2	121.00 (16)	O4—C15—H15B	109.8
O2—C1—C2	115.67 (16)	C16—C15—H15B	109.8
C3—C2—C7	121.05 (17)	H15A—C15—H15B	108.3
C3—C2—C1	120.35 (16)	C1—O2—H2	109.5
C7—C2—C1	118.59 (16)	C4—O3—C8	118.81 (14)
C2—C3—C4	119.62 (17)	C6—O4—C15	117.70 (15)
C2—C3—H3	120.2	C17—C16—C21	121.6 (8)
C4—C3—H3	120.2	C17—C16—C15	120.5 (8)
O3—C4—C5	124.69 (16)	C21—C16—C15	117.4 (4)
O3—C4—C3	115.23 (16)	C16—C17—C18	115.3 (14)
C5—C4—C3	120.07 (17)	C16—C17—H17	122.4
C4—C5—C6	119.60 (17)	C18—C17—H17	122.4
C4—C5—H5	120.2	C19—C18—C17	123.6 (17)
C6—C5—H5	120.2	C19—C18—H18	118.2
O4—C6—C7	124.09 (17)	C17—C18—H18	118.2
O4—C6—C5	114.69 (16)	C20—C19—C18	117.0 (17)
C7—C6—C5	121.21 (17)	C20—C19—H19	121.5
C6—C7—C2	118.43 (17)	C18—C19—H19	121.5
C6—C7—H7	120.8	C19—C20—C21	124.2 (14)
C2—C7—H7	120.8	C19—C20—H20	117.9
O3—C8—C9	107.77 (15)	C21—C20—H20	117.9
O3—C8—H8A	110.2	C20—C21—C16	117.1 (9)
C9—C8—H8A	110.2	C20—C21—H21	121.4
O3—C8—H8B	110.2	C16—C21—H21	121.4
C9—C8—H8B	110.2	C20A—C21A—H21A	116.9
H8A—C8—H8B	108.5	C18A—C17A—H17A	119.2
C14—C9—C10	114.7 (15)	C17A—C18A—C19A	117.3 (13)
C14—C9—C8	122.2 (8)	C17A—C18A—H18A	121.3
C10—C9—C8	123.1 (13)	C19A—C18A—H18A	121.3
C12—C11—C10	112 (3)	C20A—C19A—C18A	122.3 (15)
C12—C11—H11	124.2	C20A—C19A—H19A	118.8
C10—C11—H11	124.2	C18A—C19A—H19A	118.8
C9—C10—C11	126 (3)	C21A—C20A—C19A	116.2 (16)
C9—C10—H10	116.9	C21A—C20A—H20A	121.9
C11—C10—H10	116.9	C19A—C20A—H20A	121.9
C13—C12—C11	124 (3)	C12A—C11A—C10A	121.5 (19)
C13—C12—H12	117.9	C12A—C11A—H11A	119.3
C11—C12—H12	117.9	C10A—C11A—H11A	119.3
C12—C13—C14	118 (2)	C11A—C10A—H10A	121.4
C12—C13—H13	121.1	C11A—C12A—C13A	117 (2)
C14—C13—H13	121.1	C11A—C12A—H12A	121.6
C9—C14—C13	125.4 (14)	C13A—C12A—H12A	121.6
C9—C14—H14	117.3	C13A—C14A—H14A	120.4
C13—C14—H14	117.3	C14A—C13A—C12A	125 (2)
O4—C15—C16	109.24 (18)	C14A—C13A—H13A	117.6
O4—C15—H15A	109.8	C12A—C13A—H13A	117.6
			
O1—C1—C2—C3	179.21 (16)	C10—C9—C14—C13	1.5 (18)
O2—C1—C2—C3	−1.0 (2)	C8—C9—C14—C13	−177.8 (11)
O1—C1—C2—C7	0.4 (3)	C12—C13—C14—C9	0 (3)
O2—C1—C2—C7	−179.85 (16)	C5—C4—O3—C8	4.4 (3)
C7—C2—C3—C4	−1.0 (3)	C3—C4—O3—C8	−175.13 (16)
C1—C2—C3—C4	−179.76 (15)	C9—C8—O3—C4	173.58 (17)
C2—C3—C4—O3	−179.84 (15)	C7—C6—O4—C15	12.0 (3)
C2—C3—C4—C5	0.6 (3)	C5—C6—O4—C15	−167.82 (19)
O3—C4—C5—C6	−179.43 (16)	C16—C15—O4—C6	176.14 (18)
C3—C4—C5—C6	0.1 (3)	O4—C15—C16—C17	11.0 (9)
C4—C5—C6—O4	179.37 (17)	O4—C15—C16—C21	−161.8 (4)
C4—C5—C6—C7	−0.4 (3)	C21—C16—C17—C18	−12.9 (17)
O4—C6—C7—C2	−179.70 (18)	C15—C16—C17—C18	174.6 (9)
C5—C6—C7—C2	0.1 (3)	C16—C17—C18—C19	8 (2)
C3—C2—C7—C6	0.6 (3)	C17—C18—C19—C20	−1 (3)
C1—C2—C7—C6	179.44 (16)	C18—C19—C20—C21	−1 (3)
O3—C8—C9—C14	−114.4 (7)	C19—C20—C21—C16	−3.7 (18)
O3—C8—C9—C10	66.4 (9)	C17—C16—C21—C20	11.5 (13)
C14—C9—C10—C11	1 (2)	C15—C16—C21—C20	−175.8 (6)
C8—C9—C10—C11	179.8 (15)	C17A—C18A—C19A—C20A	−1 (3)
C12—C11—C10—C9	−3 (3)	C18A—C19A—C20A—C21A	0 (3)
C10—C11—C12—C13	4 (4)	C10A—C11A—C12A—C13A	−1 (3)
C11—C12—C13—C14	−3 (4)	C11A—C12A—C13A—C14A	−3 (4)

### Hydrogen-bond geometry (Å, º)

**Table:**

*D*—H···*A*	*D*—H	H···*A*	*D*···*A*	*D*—H···*A*
O2—H2···O1^i^	0.82	1.82	2.6333 (18)	175
C20—H20···O1^ii^	0.93	2.66	3.507 (13)	153

Symmetry codes: (i) −*x*, −*y*+1, −*z*+1; (ii) −*x*+2, −*y*, −*z*+1.
